# NAPRT loss promotes lung tumor initiation and growth through AKT signaling independently of NAD ^+^ biosynthesis

**DOI:** 10.1002/1878-0261.70330

**Published:** 2026-09-25

**Authors:** Myung Joon Oh, Ji Yun Jang, Yoon Jeon, Gi‐Cheon Kim, Hwanho Lee, Jooyoung Lee, Ju‐Hwa Kim, Hakhyun Kim, Minjee Kim, Ho‐Keun Kwon, Ho Lee, Hyun Seok Kim

**Affiliations:** ^1^ Department of Biomedical Sciences Yonsei University College of Medicine Seoul Korea; ^2^ Graduate School of Medical Science, Brain Korea 21 Project Yonsei University College of Medicine Seoul Korea; ^3^ Division of Cancer Biology, Research Institute National Cancer Center Goyang Korea; ^4^ Department of Microbiology and Immunology Yonsei University College of Medicine Seoul Korea; ^5^ Checkmate Therapeutics Inc. Seoul Korea; ^6^ Graduate Program for Nanomedical Science Yonsei University Seoul Korea; ^7^ Department of Cancer Biomedical Science National Cancer Center Graduate School of Cancer Science and Policy Goyang Korea

**Keywords:** AKT signaling, lung cancer, NAD^+^ metabolism, NAMPT dependency, NAPRT, tumor initiation

## Abstract

Nicotinamide adenine dinucleotide (NAD^+^) metabolism is frequently rewired in cancer, creating metabolic vulnerabilities that can be therapeutically exploited. Loss of nicotinate phosphoribosyltransferase (NAPRT), a rate‐limiting enzyme of the Preiss–Handler pathway, renders tumor cells dependent on nicotinamide phosphoribosyltransferase (NAMPT)‐mediated NAD^+^ synthesis and serves as a predictive biomarker for NAMPT inhibitor sensitivity. However, whether NAPRT loss actively contributes to tumorigenesis remains unclear. Here, we show that NAPRT depletion promotes tumorigenic phenotypes in lung cancer through a noncanonical mechanism independent of NAD^+^ biosynthesis. *NAPRT* knockdown enhanced clonogenic initiation, sphere formation, and invasive capacity. Mechanistically, NAPRT loss drives mTORC2‐dependent activation of the AKT signaling axis. This effect was not reversed by nicotinic acid or NAD^+^ supplementation or recapitulated by catalytic inhibition of NAPRT, indicating a function distinct from its metabolic role. Pharmacologic AKT inhibition attenuated the enhanced growth induced by NAPRT depletion. *In vivo*, lung‐specific *Naprt* deletion significantly increased tumor burden in *Kras* ^G12D^‐driven mice without altering tumor histology or immune cell composition. Together, these findings identify a previously unrecognized tumor‐suppressive role for NAPRT in lung tumor initiation and growth.

## Graphical abstract

Loss of NAPRT promotes lung tumor initiation and growth through a noncanonical mechanism, independent of its role in NAD^+^ biosynthesis. Mechanistically, NAPRT depletion activates the mTORC2‐driven AKT/β‐catenin signaling axis to enhance clonogenic and invasive phenotypes. Furthermore, lung‐specific *Naprt* deletion significantly increases tumor burden in *Kras* ^G12D^‐driven mice, highlighting its unrecognized tumor‐suppressive role. Created with BioRender.com (Agreement No. HV29ZEEYSK).

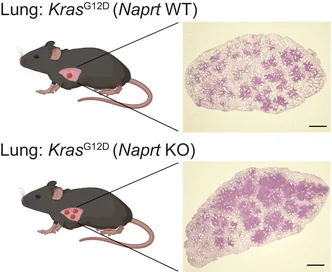

**Abbreviations**
2‐HNA: 2‐hydroxynicotinic acid
fl/fl: flox/flox
NA: nicotinic acid
NAD^+^
: Nicotinamide adenine dinucleotide
NAM: nicotinamide
NAMPT: nicotinamide phosphoribosyltransferase
NAPRT: nicotinate phosphoribosyltransferase
P/S: penicillin–streptomycin
PH pathway: Preiss–Handler pathway

## 1 Introduction

Nicotinamide adenine dinucleotide (NAD^+^) is a central metabolic cofactor and signaling substrate that supports cellular redox homeostasis, DNA repair, epigenetic regulation, and immune function [1]. In mammalian cells, NAD^+^ is maintained through three biosynthetic pathways: the *de novo* pathway from tryptophan, the Preiss–Handler (PH) pathway from nicotinic acid (NA), and the salvage pathway from nicotinamide (NAM) [2]. The rate‐limiting enzymes governing the two dominant routes are nicotinamide phosphoribosyltransferase (NAMPT), which catalyzes the salvage pathway, and nicotinate phosphoribosyltransferase (NAPRT), which mediates the PH pathway.

In multiple cancer types, epigenetic silencing or genetic loss of NAPRT renders tumor cells dependent on NAMPT‐mediated NAD^+^ synthesis. This metabolic rewiring creates a synthetic lethal vulnerability to NAMPT inhibition, establishing NAPRT deficiency as a predictive biomarker of therapeutic response [3, 4, 5].

Notably, substantial depletion of NAPRT mRNA has been observed across diverse malignancies, including colorectal, renal, hepatic, and lung cancers [3, 4, 5, 6, 7, 8]. Multiple mechanisms contribute to NAPRT suppression. In fumarate hydratase (FH)‐deficient renal cell carcinoma, accumulation of the oncometabolite fumarate induces CpG island hypermethylation within the NAPRT promoter, leading to transcriptional silencing [7]. In gliomas, PPM1D mutations disrupt activating chromatin marks (H3K4me3 and H3K27ac), whereas IDH1 mutations promote promoter methylation and transcriptional repression [9, 10]. These observations indicate that NAPRT loss arises through convergent epigenetic mechanisms across distinct tumor contexts, suggesting potential selective pressure for its depletion during tumor evolution.

Despite these findings, whether NAPRT loss is a passive consequence of malignant transformation or an active driver of tumorigenesis has remained unclear, and recent evidence has begun to favor the latter. Genetic ablation of *Naprt* was shown to accelerate tumorigenesis in both chemically induced and *Apc* ^Min/+^ colorectal cancer models [6]. Consistent with an active role, we previously demonstrated that loss of NAPRT promotes the epithelial‐to‐mesenchymal transition (EMT) and that an EMT gene expression signature correlates with reduced NAPRT expression across 942 gastric tumors, identifying NAPRT loss as a hallmark of the EMT subtype; mechanistically, NAPRT deficiency was accompanied by stabilization and accumulation of β‐catenin [4]. The molecular route linking NAPRT loss to β‐catenin accumulation, however, was not defined.

A plausible route is AKT signaling, which activates β‐catenin independently of canonical Wnt ligands. AKT promotes β‐catenin activation indirectly, by phosphorylating and inactivating GSK3β to prevent β‐catenin degradation [11], and directly, by phosphorylating β‐catenin at Ser552 to drive its nuclear accumulation and transcriptional activity [4, 12]; this cross talk between the PI3K/AKT and Wnt/β‐catenin pathways has been reviewed in the cancer setting [13]. Such activation is tumorigenically consequential: aberrant Wnt/β‐catenin activation is broadly implicated across diverse malignancies, including hepatocellular, gastric, and lung cancers [14].

Together, these observations led us to hypothesize that NAPRT loss promotes tumorigenesis by engaging the AKT/β‐catenin axis. Yet the functional evidence to date is confined to the gastrointestinal tract, and the downstream mechanisms through which NAPRT deficiency exerts its tumor‐promoting effects remain largely undefined. Here, we tested this hypothesis in lung cancer: We investigated the molecular mechanisms by which NAPRT depletion promotes tumorigenic phenotypes *in vitro* and examined the impact of NAPRT loss on tumor initiation and growth in a *Kras* ^G12D^‐driven lung tumorigenesis model.

## 2 Materials and methods

### 2.1 Spontaneous lung tumorigenesis model

This study and following animal experimental protocols were approved by the Institutional Animal Care and Use Committee (IACUC) of the National Cancer Center (NCC, approval numbers NCC‐22‐788 and NCC‐22‐789). All animal experiments were performed in strict accordance with the ethical guidelines of NCC IACUC. C57BL/6 mice were purchased from The Jackson Laboratory (Bar Harbor, ME, USA). Mice were bred and maintained under specific pathogen‐free (SPF) conditions at the Laboratory Animal Research Facility of the NCC. Genotyping was performed by PCR using the following primers: 5′‐TGC TTC CAT GTT GAG CCC ATT TTA‐3′ and 5′‐ATT CCA TGC CAG CTT TCT AGT GC‐3′. PCR products of 172 bp and 253 bp were generated for the wild‐type and floxed alleles, respectively. Six‐week‐old mice were treated with intranasal administration of an adenovirus expressing Cre recombinase (Adeno‐Cre) to induce lung tumorigenesis and *Naprt* deletion. *Naprt* ^fl/fl^; *Kras* ^G12D^ mice (*Naprt* KO) group consisted of 7 males and 2 females. *Kras* ^G12D^ control mice (*Naprt* WT) group consisted of 5 males and 4 females. Mice were euthanized 10.5 weeks after viral infection, and lungs were harvested for downstream analyses including histopathology and flow cytometry. Tumor burden was quantified by measuring the tumor area in hematoxylin and eosin (H&E)‐stained lung sections using imagej software (National Institutes of Health, Bethesda, MD, USA).

### 2.2 Immune profiling by flow cytometry

Single‐cell suspensions were prepared from harvested lung tissues. Lung tissues were minced and enzymatically digested in RPMI‐1640 medium containing collagenase II (1 mg·mL^−1^, Gibco, Grand Island, NY, USA) and DNase I (100 U·mL^−1^; Gibco) for 30 min at 37 °C. Red blood cells were removed using RBC lysis buffer (Miltenyi Biotec, Bergisch Gladbach, Germany). Cells were then stained with fluorophore‐conjugated antibodies against immune cell surface markers. Data were acquired on a BD FACSymphony A5 flow cytometer (BD Biosciences, San Jose, CA, USA) and analyzed with flowjo™ software (BD Biosciences).

### 2.3 Cell lines and cell culture

Human lung cancer cell lines (NCI‐H157 (RRID:CVCL_0463), NCI‐H1975 (RRID:CVCL_1511), NCI‐H2009 (RRID:CVCL_1514), NCI‐H2122 (RRID:CVCL_1531), and HCC4017 (RRID:CVCL_V579)) were kindly provided by Michael A. White and John D. Minna (UT Southwestern Medical Center, TX, USA). Cells were cultured in RPMI‐1640 medium supplemented with 5% fetal bovine serum (FBS; Gibco) and 1% penicillin–streptomycin (P/S; Gibco). HEK293T (RRID:CVCL_0063) and LLC1 (RRID:CVCL_4358) cells (ATCC, VA, USA) were maintained in DMEM supplemented with 10% FBS and 1% P/S. All cells were cultured at 37 °C in a humidified incubator with 5% CO_2_. Cell line identity was verified by short tandem repeat (STR) profiling performed by Macrogen (Seoul, South Korea) prior to the initiation of experiments. Mycoplasma contamination was routinely tested using the e‐Myco VALiD Mycoplasma PCR Detection Kit (LiliF Diagnostics, Gyeonggi‐do, South Korea), and all cell lines were confirmed to be mycoplasma‐free.

### 2.4 Generation of stable knockdown cell lines

For lentivirus production, 7 × 10^5^ HEK293T cells were seeded in 60‐mm dishes and cotransfected with shRNA plasmids targeting NAPRT (1 μg, TRCN0000180305) together with packaging plasmids psPAX2 (750 ng) and pMD2.G (250 ng) using FuGENE HD transfection reagent (6 μL; Promega, Madison, WI, USA). Virus‐containing media were collected 48 and 72 h after transfection. Target cells were then transduced with viral supernatants in the presence of polybrene (4 μg·mL^−1^; Cellecta, Mountain View, CA, USA). Stably transduced cells were selected with puromycin (4–10 μg·mL^−1^; Invitrogen, Carlsbad, CA, USA) for 1–2 weeks. Knockdown efficiency was validated by qPCR and western blot analysis.

### 2.5 Colony formation assay

Colony formation assay was performed as previously described [4]. Briefly, a total of 100 cells was seeded per well in 12‐well plates and cultured for 5–10 days. Colonies were washed twice with phosphate buffered saline (PBS), fixed with 100% methanol for 10 min, and stained with 0.4% crystal violet for 20 min. Colonies were imaged and quantified using imagej.

### 2.6 Invasion assay

Invasion assay was performed as previously described [4]. Briefly, a total of 5 × 10^3^ cells were seeded in the upper chamber in medium containing 0.25% FBS, while the lower chamber contained medium supplemented with 5% FBS. After 24 h, invaded cells were counted in four representative microscopic fields.

### 2.7 Sphere formation assay

Cells were seeded at a density of 5000 cells per well in ultra‐low attachment 6‐well plates. Cells were cultured in serum‐free DMEM/F12 medium supplemented with 2% B27 (Gibco), 20 ng·mL^−1^ epidermal growth factor (EGF; Gibco), and 20 ng·mL^−1^ basic fibroblast growth factor 2 (FGF2; Gibco). After 5–10 days, spheres were transferred to 96‐well plates and imaged using the Operetta CLS High‐Content Analysis System (PerkinElmer, Waltham, MA, USA). Spheres with diameters greater than 50 μm were counted.

### 2.8 Immunoblot analysis

Immunoblot analysis was performed as previously described [5]. Primary antibodies are NAPRT (1 : 1000, #NBP1‐87243; Novus Biologicals, Centennial, CO, USA), Active β‐catenin (1 : 1000, #8814; Cell Signaling Technology, Danvers, MA, USA), β‐catenin (1 : 1000, #8480; Cell Signaling Technology), phospho‐AKT (Ser473) (1 : 1000, #4060; Cell Signaling Technology), phospho‐AKT (Thr308) (1 : 1000, #4056; Cell Signaling Technology), AKT(1 : 1000, #4691; Cell Signaling Technology), HSP90 (1 : 1000, #4877; Cell Signaling Technology), phospho‐NF‐κB p65 (Ser536) (1 : 1000, #3033; Cell Signaling Technology), NF‐κB p65 (1 : 1000, #8242; Cell Signaling Technology), Rictor (1 : 1000, #2114; Cell Signaling Technology), S6 ribosomal protein (1 : 500, #2217; Cell Signaling Technology), and β‐actin (1 : 10 000, #sc‐47 778; Santa Cruz Biotechnology, Dallas, TX, USA). Immunoblots were quantified using imagej software.

### 2.9 NAD/NADH assay

NAD/NADH‐Glo™ assay kit (Promega) was used to measure NAD^+^ levels according to the manufacturer's instructions. For NA and NAD^+^ treatment experiments, cells were seeded in 96‐well culture plates. After 24 h, cells were lysed using a bicarbonate base buffer (100 mm sodium carbonate, 20 mm sodium bicarbonate, 10 mm nicotinamide, 0.05% Triton® X‐100) with 1% DTAB. To specifically measure NAD^+^, NADH was degraded by adding 0.4 N HCl, followed by neutralization with a Tris base. The NAD^+^ concentration was then measured using the NAD/NADH‐Glo™ detection reagent.

### 2.10 cDNA transfection

The pCMV6‐GFP‐NAPRT cDNA construct (#RG204356; OriGene, Rockville, MD, USA) was used for transfection. A catalytically inactive NAPRT cDNA mutant was generated via a subcloning service (CosmoGENETECH, Seoul, South Korea). For NAPRT reintroduction experiments, at 48‐h post‐siRNA transfection (pooled 5′‐GACUGGAAACAACACGAAU‐3′, 5′‐UUCGUGUUGUUUCCAGUCA‐3′, and 5′‐AAAAGUGAGUGAUUCGUGU‐3′), cells were transfected with either an empty vector or the NAPRT cDNA using Lipofectamine 2000 (Invitrogen) according to the manufacturer's instructions. Cells were harvested 48‐h post‐transfection for immunoblot analysis.

### 2.11 Cell viability assay

Cell viability assays were conducted using the CellTiter‐Glo® assay system (Promega), according to the manufacturer's instructions. Briefly, cells were seeded on the 96 wells and treated with inhibitors for 72 h. After incubation, 10 μL of CellTiter‐Glo® reagent was applied to the wells and vortexed briefly for lysis. After 15 min of incubation at room temperature, luminescence was measured with PerkinElmer Envision Multimode Plate Reader (PerkinElmer).

### 2.12 Quantitative real‐time PCR (qPCR)

Total RNA was extracted using the RNeasy Mini Kit (Qiagen, Venlo, Netherlands) according to the manufacturer's protocols. RNA concentrations were measured using a NanoDrop spectrophotometer (Thermo Fisher Scientific, Waltham, MA, USA), and 1 μg of RNA was reverse‐transcribed using PrimeScript RT Master Mix (Takara, Kyoto, Japan). qPCR was performed using SYBR Green Master Mix (Enzynomics, Daejeon, South Korea) on a QuantStudio 3 real‐time PCR System (Applied Biosystems, Waltham, MA, USA). Relative gene expression was calculated using the ΔΔ*C*_t_ method and normalized to *Gapdh* and *Actb*. The following primers were used for qPCR: *Gapdh*, 5′‐CAT CAC TGC CAC CCA GAA GAC TG‐3′ and 5′‐ATG CCA GTG AGC TTC CCG TTC AG‐3′; *Actb*, 5′‐CAT TGC TGA CAG GAT GCA GAA GG–3′ and 5′‐TGC TGG AAG GTG GAC AGT GAG G–3′; *Naprt*, 5′‐AGC TTG GCT ACA GAG CAG TG–3′ and 5′‐GCA GTT CGA AAG ATG CCA CG–3′.

### 2.13 Immunohistochemistry (IHC) analysis

Tissue sections were deparaffinized in xylene and rehydrated through a graded ethanol series. Antigen retrieval was performed by incubating the sections with Proteinase K (#S3020; Dako, Santa Clara, CA, USA) for 10 min. Endogenous peroxidase was quenched using 3% hydrogen peroxide (#3059, Duksan, Ulsan, Korea) for 10 min, followed by washing in Tris‐buffered saline (TBS). The sections were then incubated with a primary antibody with optimized concentration for 1 h at room temperature. After washing with TBS, the slides were incubated with an HRP‐labeled polymer anti‐rabbit secondary antibody (#K4003; Dako) for 20 min at room temperature. Immunoreactivity was visualized using a DAB substrate kit (#K3468; Dako). Finally, the sections were counterstained with hematoxylin for 10 min, washed, dehydrated through a graded ethanol series, cleared in xylene, and mounted. The primary antibodies are Ki‐67 (1 : 100, ab15580; Abcam, Cambridge, UK) and cleaved caspase‐3 (1 : 100, AF835; R&D Systems, Minneapolis, MN, USA). Images and quantification were analyzed by Vectra^®^ PolarisTM Automated Quantitative Pathology Imaging System with inform software (Akoya Biosciences, Marlborough, MA, USA) and QuPath v0.7.0 (University of Edinburgh, Edinburgh, United Kingdom).

### 2.14 Statistical analysis

All quantitative data are presented as mean ± standard deviation (SD) unless otherwise specified. Statistical analyses were performed using r software v4.5.2. Unless otherwise specified, experiments were performed in triplicate. Comparisons between two groups were conducted using two‐tailed Student's *t*‐test. A *P*‐value < 0.05 was considered statistically significant.

## 3 Results

### 3.1 NAPRT knockdown enhances clonogenic initiation and invasion in lung cancer cells

**Fig. 1:**
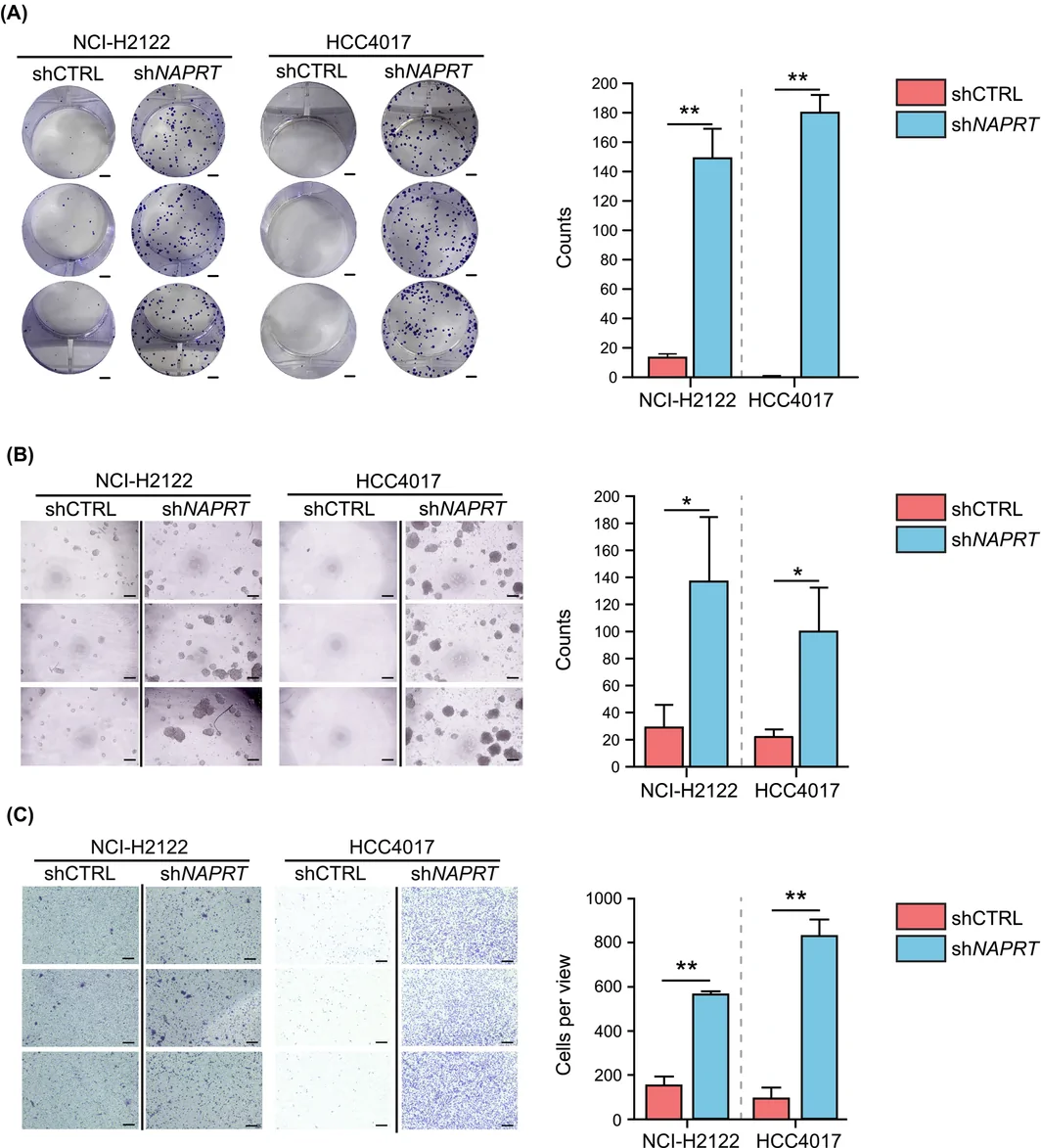
NAPRT depletion enhances clonogenic growth and invasive capacity in lung cancer cells. (A) Colony formation assay comparing control (shCTRL) and NAPRT knockdown (sh*NAPRT*) lung cancer cells under low‐density conditions. Scale bar, 2 mm. Quantification of colony numbers is shown on the right. (B) Sphere formation assay comparing shCTRL and sh*NAPRT* cells under nonadherent culture conditions. Scale bar, 50 μm. Quantification of sphere numbers is shown on the right. (C) Matrigel invasion assay assessing invasive capacity of shCTRL and sh*NAPRT* cells. Scale bar, 100 μm. Quantification of invaded cells is shown on the right. (A–C) Data are presented as mean ± SD (*n* = 3 per group). Statistical significance was determined using a two‐tailed Student's *t*‐test. *P < 0.05, **P < 0.01.

We generated stable NAPRT knockdown (sh*NAPRT*) lung cancer cell lines to assess the functional role of NAPRT in cancer. Compared with control cells (shCTRL), sh*NAPRT* cells displayed a significant increase in colony formation under highly diluted seeding conditions (Fig. 1A), indicating enhanced clonogenic initiation capacity. In parallel, sh*NAPRT* cells exhibited increased sphere formation under nonadherent, serum‐free conditions, consistent with augmented clonogenic growth in sphere culture (Fig. 1B). Moreover, Matrigel transwell assays revealed that *NAPRT* knockdown markedly enhanced invasive behavior (Fig. 1C). Together, these findings indicate that loss of *NAPRT* promotes both clonal growth initiation and invasive capacity, cellular properties associated with aggressive tumor phenotypes.

### 3.2 NAPRT loss activates AKT/β‐catenin signaling to promote clonogenic growth independently of NAD ^+^ biosynthesis

**Fig. 2:**
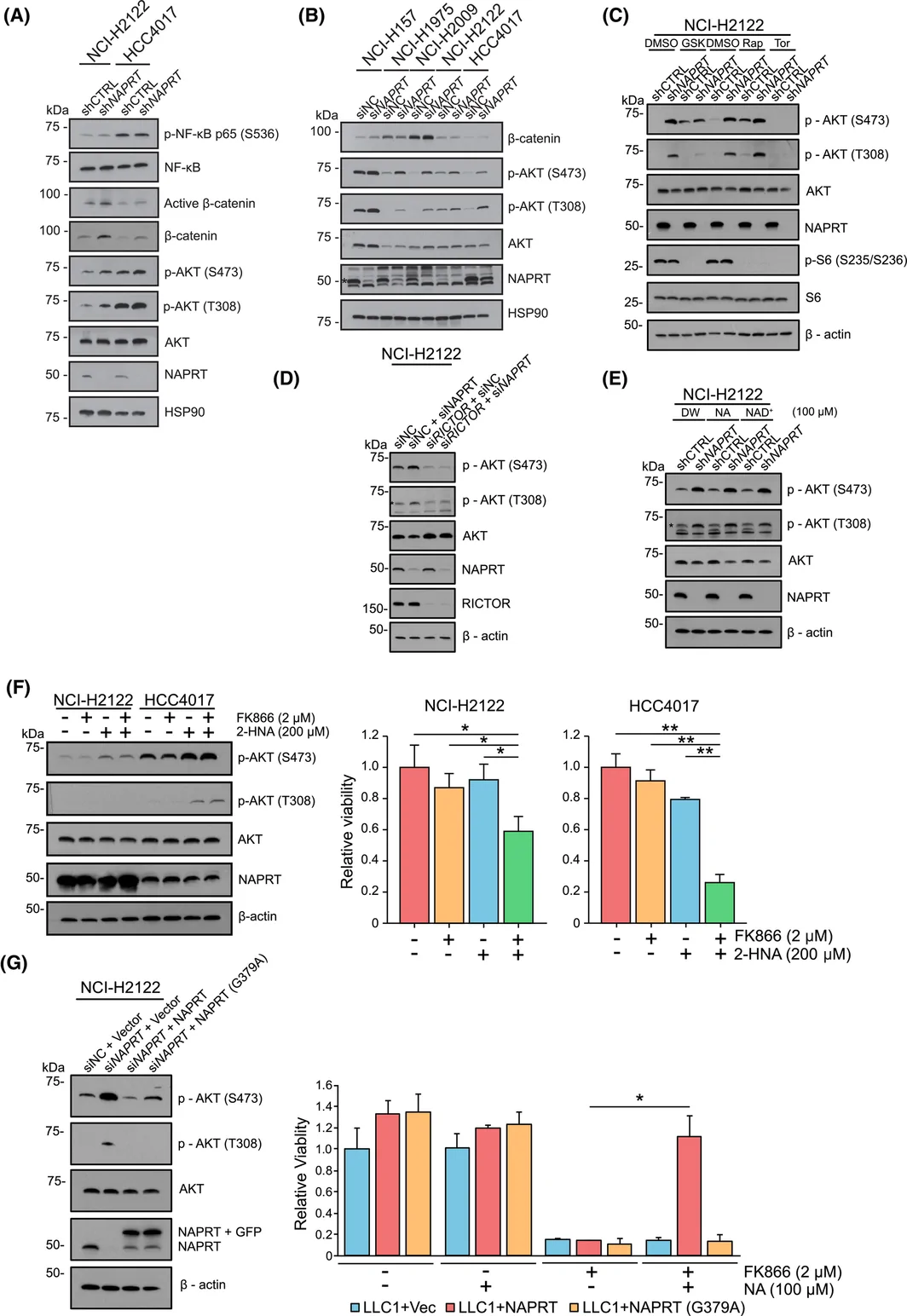
NAPRT depletion activates AKT/β‐catenin signaling independently of NAD^+^ biosynthesis. (A) Immunoblot analysis of NF‐κB and PI3K/AKT pathway proteins in control (shCTRL) and NAPRT knockdown (sh*NAPRT*) lung cancer cells. (B) Immunoblot analysis of AKT phosphorylation and β‐catenin accumulation following transient NAPRT knockdown (siRNA) in lung cancer cells. (C) Immunoblot analysis of NCI‐H2122 cells treated with the PDK1 inhibitor GSK2334470 (GSK; 1 μm, 6 h), the mTORC1 inhibitor Rapamycin (Rap; 100 nm, 12 h), or the dual mTORC1/mTORC2 inhibitor Torin 1 (Tor; 250 nm, 12 h). (D) Immunoblot analysis of NCI‐H2122 cells treated with siRNAs against NAPRT or RICTOR. (E) Immunoblot analysis of NCI‐H2122 cells treated with nicotinic acid (NA, 100 μm) or NAD^+^ (100 μm) for 24 h. (F) Left: Immunoblot analysis of lung cancer cells treated with the NAPRT inhibitor 2‐hydroxynicotinic acid (2‐HNA, 200 μm) and the NAMPT inhibitor FK866 (2 μm) for 72 h. Right: The on‐target effect of 2‐HNA is shown by enhanced cytotoxicity in combination with FK866. (G) Left: Immunoblot analysis of NCI‐H2122 cells following siRNA‐mediated NAPRT knockdown and re‐expression of siRNA‐resistant wild‐type or catalytically inactive (G379A) NAPRT. Right: Catalytic inactivity was confirmed in LLC1 cells, which lack basal NAPRT expression; cells survived FK866 only when expressing catalytically active NAPRT in the presence of NA. (A–G) All immunoblots are representative images from one of three independent experiments with similar results, except the immunoblot in (G, left), which was performed once. (F, G) Data are presented as mean ± SD (*n* = 3 per group). Statistical significance was determined using a two‐tailed Student's *t*‐test. *P < 0.05, **P < 0.01.

In our previous work, re‐expression of NAPRT in NAPRT‐deficient gastric cancer cells reduced β‐catenin protein stability, whereas NAPRT depletion increased it [4]. AKT has been reported to promote β‐catenin activation either indirectly, by inhibiting GSK3β (thereby preventing β‐catenin degradation), or directly, by phosphorylating β‐catenin at Ser552 to enhance its nuclear accumulation and transcriptional activity [4, 11, 12, 13]. Based on the accumulation of β‐catenin observed in lung cancer cells upon NAPRT loss, we hypothesized that this effect might be mediated by activation of the upstream AKT signaling axis. Consistent with this model, immunoblotting revealed that stable knockdown of NAPRT robustly increased AKT phosphorylation at the activating residues Ser473 (S473) and Thr308 (T308), accompanied by accumulation of total β‐catenin protein (Fig. 2A). This phenotype was consistently observed across multiple lung cancer cell lines following transient siRNA‐mediated knockdown of NAPRT (Fig. 2B).

To define the mechanism linking NAPRT loss to AKT activation, we examined the kinase dependency of the elevated AKT phosphorylation. Since p‐AKT (T308) and p‐AKT (S473) are phosphorylated by PDK1 and mTORC2, respectively, NAPRT‐depleted cells were treated with the selective PDK1 inhibitor GSK2334470, the mTORC1 inhibitor rapamycin, or the dual mTORC1/mTORC2 inhibitor Torin 1 (Fig. 2C). PDK1 inhibition selectively reduced p‐AKT (T308) while leaving p‐AKT (S473) largely unchanged. Rapamycin did not normalize phosphorylation at either site, indicating that mTORC1 is not responsible for the effect. In contrast, Torin 1 restored both p‐AKT (T308) and p‐AKT (S473) to basal levels (Fig. 2C). Because mTORC2 is the kinase distinguishing Torin 1 from rapamycin, these results identify mTORC2 as the principal driver of NAPRT loss‐induced AKT activation. Consistent with this, genetic codepletion of RICTOR, a core mTORC2 component, together with NAPRT reversed AKT phosphorylation at both sites, phenocopying Torin 1 treatment (Fig. 2D). Taken together, these data demonstrate that NAPRT loss promotes AKT phosphorylation through an mTORC2‐dependent mechanism.

To determine whether activation of the AKT/β‐catenin axis depended on the canonical enzymatic function of NAPRT in NAD^+^ biosynthesis, we modulated intracellular NAD^+^ availability through multiple approaches. Supplementation with NAD^+^—the end product of the NAPRT‐mediated Preiss–Handler pathway—at a concentration that sufficiently elevates intracellular NAD^+^ levels in sh*NAPRT* cells (Fig. S1A,B) did not reverse the elevated AKT phosphorylation observed in these cells (Fig. 2E). Although NAPRT knockdown blocks the Preiss–Handler pathway and therefore precludes NAD^+^ synthesis from nicotinic acid (NA), we additionally supplemented NA to control for any effect arising from intracellular NA accumulation upon NAPRT loss; this supplementation did not increase AKT phosphorylation in NAPRT‐intact control cells (Fig. 2E), excluding NA accumulation as a contributing factor. Moreover, chemical inhibition of NAPRT catalytic activity using 2‐hydroxynicotinic acid (2‐HNA), at concentrations that synergistically reduced cell viability in combination with the NAMPT inhibitor FK866 (Fig. 2F, right), failed to induce AKT phosphorylation (Fig. 2F, left; Fig. S2). To corroborate these findings using a complementary genetic approach, we next performed rescue experiments with a catalytically inactive NAPRT mutant (G379A) in si*NAPRT* cells [15]. Consistent with a loss of enzymatic activity, re‐expression of wild‐type NAPRT, but not the G379A mutant, rescued si*NAPRT* cells from FK866‐induced NAD^+^ depletion, confirming that G379A is catalytically inactive (Fig. 2G, right). Despite this loss of NAD^+^ biosynthetic activity, re‐introduction of both wild‐type and catalytically inactive (G379A) NAPRT suppressed AKT phosphorylation to a comparable extent (Fig. 2G, left). Together with the metabolite supplementation and pharmacological inhibition experiments described above, these results indicate that NAPRT suppression activates AKT/β‐catenin signaling through a mechanism that is independent of its canonical catalytic function in NAD^+^ biosynthesis.

### 3.3 AKT inhibition attenuates NAPRT loss‐induced clonogenic growth in lung cancer cells

**Fig. 3:**
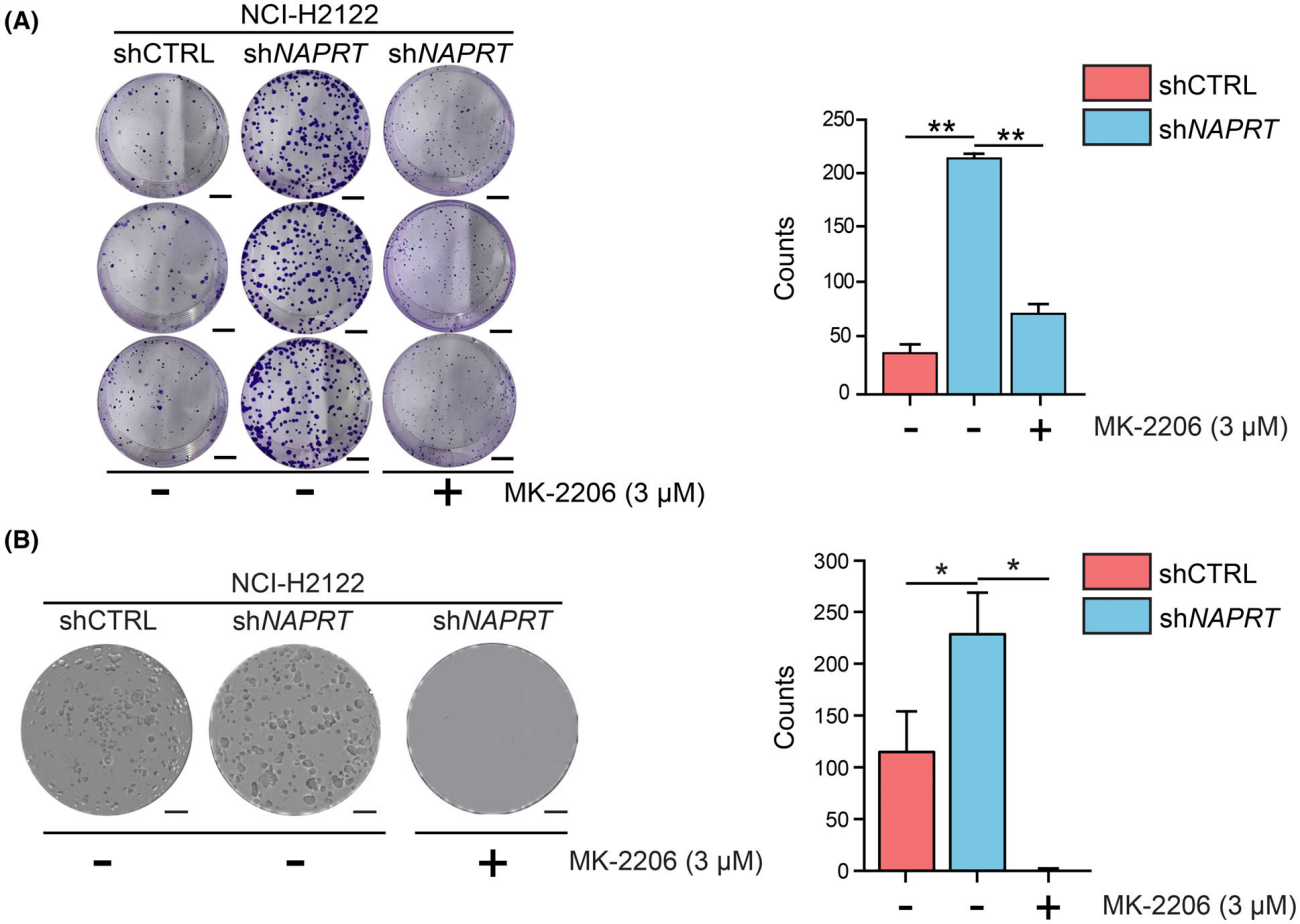
AKT inhibition suppresses NAPRT loss‐induced clonogenic growth in lung cancer cells. (A) Colony formation assay comparing control (shCTRL) and NAPRT knockdown (sh*NAPRT*) lung cancer cells. Cells were treated with MK‐2206 (3 μm) for 72 h. Scale bar, 2 mm. Quantification is shown on the right. (B) Sphere formation assay comparing shCTRL and sh*NAPRT* cells following treatment with MK‐2206 (3 μm) for 72 h. Scale bar, 1 mm. Quantification is shown on the right. (A, B) Data are presented as mean ± SD (*n* = 3 per group). Statistical significance was determined using a two‐tailed Student's *t*‐test. *P < 0.05, **P < 0.01.

To determine whether the protumorigenic phenotypes induced by NAPRT loss depend on AKT hyperactivation, NAPRT knockdown cells were treated with MK‐2206, a selective allosteric AKT inhibitor [16]. MK‐2206 treatment significantly reduced the enhanced colony formation observed under low‐density conditions in NAPRT‐deficient cells (Fig. 3A). Similarly, MK‐2206 markedly suppressed sphere formation under nonadherent conditions (Fig. 3B).

These findings indicate that AKT activation is required for the enhanced clonogenic growth associated with NAPRT loss.

### 3.4 Lung‐specific *Naprt* deletion increases tumor burden in a *Kras* ^G12D^ ‐driven lung cancer model

**Fig. 4:**
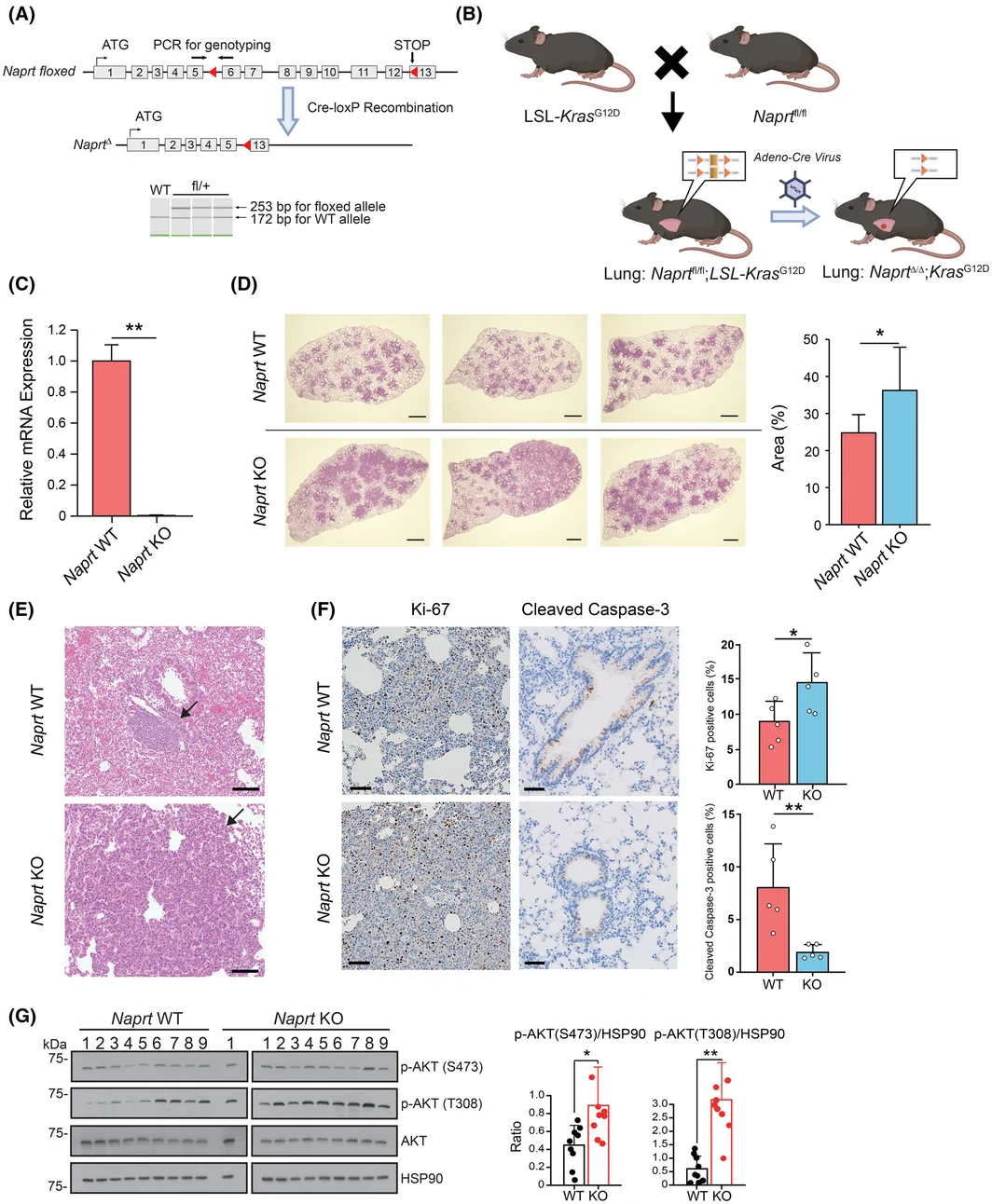
*Naprt* deletion increases tumor burden in a *Kras* ^G12D^‐driven mouse lung cancer model. (A) Schematic representation of the *Naprt* floxed allele and Cre‐mediated recombination. Representative PCR genotyping of *Naprt* floxed mice is shown at the bottom. This schematic was created using microsoft powerpoint. (B) Experimental strategy for lung‐specific *Naprt* deletion in a *Kras* ^G12D^‐driven lung cancer model. This schematic was created with BioRender.com (Agreement No. HV29ZEEYSK). (C) Relative *Naprt* mRNA levels in lungs from *Naprt* wild‐type (WT) and knockout (KO) *Kras* ^G12D^ mice. (D) Left, representative images of lungs harvested 10.5 weeks after Adeno‐Cre‐mediated tumor induction. Scale bar, 2 mm. Right, quantification of lung tumor burden in *Naprt* WT and *Naprt* KO mice. (E) Representative hematoxylin and eosin (H&E)‐stained histological images of lung tumors from *Naprt* WT and *Naprt* KO mice (*n* = 9 mice per group; one representative image per group is shown). Similar histological features were observed in all mice examined. Arrow indicates a proliferative lesion. Scale bar, 200 μm. (F) Representative immunohistochemistry (IHC) images of Ki‐67 and cleaved caspase‐3 staining in lung tumors from *Naprt* WT and *Naprt* KO mice. Quantification of DAB‐positive cells is shown on the right (*n* = 5 per group; mean ± SEM; two‐tailed Student's *t*‐test). Scale bar, 50 μm. (G) Immunoblot analysis of lung lysates from *Kras* ^G12D^ mice showing increased AKT phosphorylation by *Naprt* deletion. Quantification is shown on the right. Band intensities of phospho‐AKT were first normalized to HSP90 and then further normalized across blots using the matched control sample (#1) within each blot. (C, D, G) Data are presented as mean ± SD (*n* = 9 per group). Statistical significance was determined using a two‐tailed Student's *t*‐test. *P < 0.05, **P < 0.01.

To investigate the function of NAPRT in lung tumorigenesis *in vivo*, we employed genetically engineered C57BL/6 mice carrying a conditional *Kras* ^G12D^ allele and floxed *Naprt* alleles (*Naprt* ^fl/fl^). For this, *Naprt* conditional knockout mice were generated using the CRISPR/Cas9 technology on a C57BL/6 background. Two loxP sites were inserted flanking exons 6–13 of the *Naprt* gene to enable conditional deletion (Fig. 4A). Tumors were induced by intranasal administration of Adeno‐Cre virus (Fig. 4B), and efficient deletion of *Naprt* in lung tissue was confirmed by qPCR (Fig. 4C). Mice were monitored for 10.5 weeks following viral infection, after which all animals were euthanized for analysis (Fig. 4D).

Histopathological examination of lung sections revealed a significant increase in total tumor area in *Naprt* ^fl/fl^; *Kras* ^G12D^ mice (*Naprt* KO) compared with *Kras* ^G12D^ control mice (Fig. 4D). Tumors in both genotypes were predominantly adenomas, with no evidence of progression to adenocarcinoma at the humane endpoint (10.5‐week postinduction; Fig. 4E), indicating that *Naprt* deletion increases tumor initiation and growth without overtly affecting histological progression in this oncogenic *Kras*‐driven lung tumor model. To determine whether the increased proliferative lesions in the *Naprt* KO lung reflect enhanced proliferation, reduced apoptosis, or both, we performed immunohistochemistry for Ki‐67 and cleaved caspase‐3. Lesions in the KO lung showed significantly higher Ki‐67 positivity and lower cleaved caspase‐3 positivity than controls (Fig. 4F), indicating that the increased lesion burden is attributable to both elevated proliferation and reduced apoptosis.

Flow cytometric analysis of immune cells within the tumor immune microenvironment (TIME) revealed no significant differences in the proportions of major immune cell populations between *Naprt* KO and *Kras* ^G12D^ control mice (Fig. S3). Western blot analysis of mice lungs showed increased AKT phosphorylation in *Naprt* KO groups (Fig. 4G). These findings suggest that the increased tumor burden associated with *Naprt* loss is predominantly driven by cancer cell‐intrinsic mechanisms. Although no major changes in immune composition were observed, a contribution from subtle functional alterations in immune cells or cytokine signaling cannot be entirely excluded.

## 4 Discussion

In this study, we identify a previously unappreciated, noncanonical role of NAPRT in restraining clonogenic growth and tumor expansion in lung cancer. Loss of NAPRT enhanced clonogenic initiation, invasive behavior, and tumor burden in a *Kras*‐driven lung cancer model, effects that were mediated by activation of the AKT/β‐catenin axis and occurred independently of NAPRT's canonical function in NAD^+^ biosynthesis.

Our findings extend prior work by demonstrating that NAPRT regulates tumor‐associated signaling independently of its enzymatic activity in the Preiss–Handler pathway. Three lines of evidence argue against a purely metabolic mechanism: Neither NAD^+^ modulation nor pharmacologic NAPRT inhibition recapitulated AKT activation, and re‐expression of a catalytically inactive NAPRT mutant was sufficient to reverse AKT hyperphosphorylation. Building on this, our inhibitor and RICTOR‐depletion experiments place NAPRT loss upstream of mTORC2‐driven AKT activation. How NAPRT loss is sensed and relayed to the mTORC2–AKT axis remains to be determined and represents an important direction for future study.

Previous studies have reported an association between NAPRT loss and activation of epithelial–mesenchymal transition (EMT)‐related programs in cancer [4, 5]. In this context, our findings provide mechanistic and functional insights that complement these observations. Loss of NAPRT enhanced invasive behavior and clonogenic growth, phenotypes commonly associated with partial or hybrid EMT states, and was accompanied by activation of the AKT/β‐catenin axis, a signaling pathway known to cooperate with EMT‐associated transcriptional programs [17]. Emerging evidence suggests that epithelial–mesenchymal programs exist on a continuum, including partial EMT states that can be identified even in early tumorigenic lesions such as hyperplasia and adenoma [18]. Such partial EMT phenotypes have been associated with increased cellular plasticity, invasion, and clonogenic potential without full mesenchymal conversion, providing a conceptual framework for interpreting our findings.

Although prior genetic loss‐of‐function studies had established a causal role for NAPRT in the EMT program, the supporting evidence was derived exclusively from nonphysiological, reductionist systems—*in vitro* cell lines and xenografts—that do not recapitulate the native tissue environment, and the impact of NAPRT loss on tumor initiation and proliferation had not been examined. To overcome these limitations, we established lung‐specific *Naprt* deletion in the autochthonous LSL‐*Kras* ^G12D^ model, enabling interrogation of Naprt function in a physiologically intact, tissue‐specific context. To our knowledge, this provides the first *in vivo* genetic evidence that lung‐specific *Naprt* loss modulates *Kras*‐driven tumor initiation and proliferation within a native lung environment—a function distinct from the previously characterized EMT phenotype and not addressable in prior cell line, transcriptomic, or implantation‐based studies.

*In vivo*, lung‐specific deletion of *Naprt* significantly increased tumor burden in a *Kras* ^G12D^‐driven model without altering histological grade or immune cell composition. Establishment of lung‐specific deletion of *Naprt* in the LSL‐*Kras* ^G12D^ mouse model led to elucidation of the effect of Naprt loss exclusively in the lung, excluding the effect of Naprt loss in the other parts of the body. The increased tumor burden observed in *Naprt*‐deficient, *Kras*‐driven lung tumors likely reflects enhanced clonal growth and survival of tumor cells in early‐stage lesions, rather than accelerated progression to invasive carcinoma. In this setting, EMT‐associated cellular plasticity may facilitate tumor expansion and adaptability while preserving an epithelial architecture sufficient to maintain adenomatous histology. Moreover, the lack of progression to adenocarcinoma during the observation period suggests that additional genetic or microenvironmental events may be required for full malignant conversion, and that Naprt loss alone is insufficient to drive late‐stage progression in this *Kras*‐driven lung cancer model.

This study has several limitations. The precise molecular mechanism linking NAPRT loss to AKT activation remains to be elucidated, and further studies will be required to define whether AKT/β‐catenin‐dependent transcriptional programs are functionally engaged *in vivo*. Furthermore, future studies are warranted to determine whether subtle, noncompositional changes in the immune microenvironment—such as altered immune cell function or cytokine signaling—contribute to the phenotype observed following NAPRT loss.

Together, our findings suggest that NAPRT status may influence tumor behavior through signaling pathways independent of NAD^+^ metabolism, with potential implications for therapeutic strategies that target NAD^+^ biosynthesis in cancer.

## Conflict of interest

H.S.K. is a founder and chief scientific officer of Checkmate Therapeutics Inc. The authors have no other competing or financial conflicts of interest.

## Author contributions

HSK contributed to conceptualization; MJO, G‐CK, HwL, J‐HK, HK, and MK contributed to investigation; JYJ, YJ, JL, HoL, and H‐KK contributed to resources and methodology; MJO contributed to writing – original draft; HSK contributed to writing – review and editing; HSK, HoL, and H‐KK contributed to supervision.

## Supporting information


**Fig. S1.** Exogenous NAD^+^ supplementation increases intracellular NAD^+^ concentration and rescues cell viability in NAPRT‐depleted cells.
**Fig. S2.** Quantification of p‐AKT (Ser473)/AKT and p‐AKT (Thr308)/AKT ratios from the immunoblot shown in Fig. 2F.
**Fig. S3.** Flow cytometry analysis of immune cell population in lung tissues showed no significant differences.

## Data Availability

All data supporting the findings of this study are provided within the paper and its Supporting Information. Raw data underlying the reported findings are available from the corresponding authors upon reasonable request.

## References

[1] Covarrubias AJ , Perrone R , Grozio A , Verdin E . NAD+ metabolism and its roles in cellular processes during ageing. Nat Rev Mol Cell Biol. 2021;22(2):119–141. 10.1038/s41580-020-00313-x PMC7963035 33353981

[2] Navas LE , Carnero A . NAD+ metabolism, stemness, the immune response, and cancer. Signal Transduct Target Ther. 2021;6(1):2. 10.1038/s41392-020-00354-w PMC7775471 33384409

[3] Shames DS , Elkins K , Walter K , Holcomb T , Du P , Mohl D , et al. Loss of NAPRT1 expression by tumor‐specific promoter methylation provides a novel predictive biomarker for NAMPT inhibitors. Clin Cancer Res. 2013;19(24):6912–6923. 10.1158/1078-0432.CCR-13-1186 24097869

[4] Lee J , Kim H , Lee JE , Shin S‐J , Oh S , Kwon G , et al. Selective cytotoxicity of the NAMPT inhibitor FK866 toward gastric cancer cells with markers of the epithelial‐mesenchymal transition, due to loss of NAPRT. Gastroenterology. 2018;155(3):799–814. 10.1053/j.gastro.2018.05.024 29775598

[5] Kim M , Kim H , Kang B‐G , Lee J , Kim T , Lee H , et al. Discovery of a novel NAMPT inhibitor that selectively targets NAPRT‐deficient EMT‐subtype cancer cells and alleviates chemotherapy‐induced peripheral neuropathy. Theranostics. 2023;13(14):5075–5098. 10.7150/thno.85356 PMC10526665 37771778

[6] Wu X , Williams JG , Liang H , Gruzdev A , Hartsell J , Shpargel J , et al. NAPRT‐mediated deamidated NAD biosynthesis enhances colon tissue resiliency and suppresses tumorigenesis. Nat Commun. 2026;17(1):8651. 10.1038/s41467-026-68998-w PMC13490428 41667489

[7] Noronha KJ , Lucas KN , Paradkar S , Edmonds J , Friedman S , Murray MA , et al. NAPRT silencing in FH‐deficient renal cell carcinoma confers therapeutic vulnerabilities via NAD+ depletion. Mol Cancer Res. 2024;22(10):973–988. 10.1158/1541-7786.MCR-23-1003 PMC11445649 38949523

[8] Zhu X , Li Y , Liu H , Wang Y , Sun R , Jiang Z , et al. NAMPT‐targeting PROTAC and nicotinic acid co‐administration elicit safe and robust anti‐tumor efficacy in NAPRT‐deficient pan‐cancers. Cell Chem Biol. 2024;31(6):1203–1218. 10.1016/j.chembiol.2024.05.007 38906111

[9] Fons NR , Sundaram RK , Breuer GA , Peng S , McLean RL , Kalathil AN , et al. PPM1D mutations silence NAPRT gene expression and confer NAMPT inhibitor sensitivity in glioma. Nat Commun. 2019;10(1):3790. 10.1038/s41467-019-11732-6 PMC6706443 31439867

[10] Tateishi K , Wakimoto H , Iafrate AJ , Tanaka S , Loebel F , Lelic N , et al. Extreme vulnerability of IDH1 mutant cancers to NAD+ depletion. Cancer Cell. 2015;28(6):773–784. 10.1016/j.ccell.2015.11.006 PMC4684594 26678339

[11] Sharma M , Chuang WW , Sun Z . Phosphatidylinositol 3‐kinase/Akt stimulates androgen pathway through GSK3β inhibition and nuclear β‐catenin accumulation. J Biol Chem. 2002;277(34):30935–30941. 10.1074/jbc.M201919200 12063252

[12] Fang D , Hawke D , Zheng Y , Xia Y , Meisenhelder J , Nika H , et al. Phosphorylation of beta‐catenin by AKT promotes beta‐catenin transcriptional activity. J Biol Chem. 2007;282(15):11221–11229. 10.1074/jbc.M611871200 PMC1850976 17287208

[13] Hassanein EHM , Althagafy HS , Abd‐ElHafeez HH , Ibrahim IM , Alghamdi BS . Harnessing GSK‐3β inhibition for lung cancer therapy: emerging opportunities and challenges. Med Oncol. 2025;42(12):548. 10.1007/s12032-025-03086-5 PMC12605635 41217667

[14] Yu F , Yu C , Li F , Zuo Y , Wang Y , Yao L , et al. Wnt/β‐catenin signaling in cancers and targeted therapies. Signal Transduct Target Ther. 2021;6(1):307. 10.1038/s41392-021-00701-5 PMC8403677 34456337

[15] Managò A , Audrito V , Mazzola F , Sorci L , Gaudino F , Gizzi K , et al. Extracellular nicotinate phosphoribosyltransferase binds toll like receptor 4 and mediates inflammation. Nat Commun. 2019;10(1):4116. 10.1038/s41467-019-12055-2 PMC6739309 31511522

[16] Hirai H , Sootome H , Nakatsuru Y , Miyama K , Taguchi S , Tsujioka K , et al. MK‐2206, an allosteric Akt inhibitor, enhances antitumor efficacy by standard chemotherapeutic agents or molecular targeted drugs in vitro and in vivo. Mol Cancer Ther. 2010;9(7):1956–1967. 10.1158/1535-7163.MCT-09-1012 20571069

[17] Lamouille S , Xu J , Derynck R . Molecular mechanisms of epithelial‐mesenchymal transition. Nat Rev Mol Cell Biol. 2014;15(3):178–196. 10.1038/nrm3758 PMC4240281 24556840

[18] Lüönd F , Sugiyama N , Bill R , Bornes L , Hager C , Tang F , et al. Distinct contributions of partial and full EMT to breast cancer malignancy. Dev Cell. 2021;56(23):3203–3221. 10.1016/j.devcel.2021.11.006 34847378

