# Supplementary material for: NAPRT loss promotes lung tumor initiation and growth through AKT signaling independently of NAD + biosynthesis
Source: Mol Oncol. 2026 Sep 25:10.1002/1878-0261.70330. Online ahead of print. doi: 10.1002/1878-0261.70330 (PMC13613957; doi:10.1002/1878-0261.70330)
Supplement: Supplementary file 1 [file MOL2-9999-0-s001.docx]

**NAPRT loss promotes lung tumor initiation and growth through AKT signaling independently of NAD^+^ biosynthesis**

**Supplementary Figures**

**Figure S1: Exogenous NAD⁺ supplementation increases intracellular NAD⁺ concentration and rescues cell viability in NAPRT-depleted cells.**

(A) Relative intracellular NAD⁺ concentration in NCI-H2122-shNAPRT cells treated with distilled water (DW; vehicle) or exogenous NAD⁺ (50 μM, 100 μM) for 24 h. Data are presented as mean ± SD (n = 4 per group). Statistical significance was determined by two-tailed Student's t-test. *p < 0.05, **p < 0.01.

(B) Relative viability of NCI-H2122-shCTRL (DW only) and NCI-H2122-shNAPRT cells (DW or exogenous NAD⁺ [50 μM, 100 μM]) treated with FK866 (2 μM) for 72 h. Data are presented as mean ± SD (n = 3 per group). Statistical significance was determined by two-tailed Student's t-test. **p < 0.01.

**Figure S2: Quantification of p-AKT (Ser473)/AKT and p-AKT (Thr308)/AKT ratios from the immunoblot shown in Figure 2F.**

Densitometric quantification was performed using ImageJ. Data are presented as mean ± SD (n = 3 per group). The effect of 2-HNA treatment was assessed by two-tailed Student's t-test (pairwise comparisons between 2-HNA-treated and untreated cells within each FK866 condition, for both cell lines); no statistically significant differences were detected. ns, not significant.

**Figure S3: Flow cytometry analysis of immune cell population in lung tissues showed no significant differences.**

Flow cytometry analysis of immune cell populations in lung tissues from *Naprt* WT and *Naprt* KO mice. Data are presented as mean ± SEM (n = 5 or 7 per group). Statistical significance was determined using a two-tailed Student's t-test; no statistically significant differences were detected. ns, not significant.
